# Influence of Aerosol Chemical Composition on Condensation Sink Efficiency and New Particle Formation in Beijing

**DOI:** 10.1021/acs.estlett.2c00159

**Published:** 2022-04-12

**Authors:** Wei Du, Jing Cai, Feixue Zheng, Chao Yan, Ying Zhou, Yishuo Guo, Biwu Chu, Lei Yao, Liine M. Heikkinen, Xiaolong Fan, Yonghong Wang, Runlong Cai, Simo Hakala, Tommy Chan, Jenni Kontkanen, Santeri Tuovinen, Tuukka Petäjä, Juha Kangasluoma, Federico Bianchi, Pauli Paasonen, Yele Sun, Veli-Matti Kerminen, Yongchun Liu, Kaspar R. Daellenbach, Lubna Dada, Markku Kulmala

**Affiliations:** † Aerosol and Haze Laboratory, Beijing Advanced Innovation Center for Soft Matter Science and Engineering, Beijing University of Chemical Technology, Beijing 100089, China; ‡ Institute for Atmospheric and Earth System Research/Physics, Faculty of Science, University of Helsinki, Helsinki 00014, Finland; § State Key Joint Laboratory of Environment Simulation and Pollution Control, Research Center for Eco-Environmental Sciences, Chinese Academy of Sciences, Beijing 100085, China; ∥ State Key Laboratory of Atmospheric Boundary Layer Physics and Atmospheric Chemistry, Institute of Atmospheric Physics, Chinese Academy of Sciences, Beijing 100029, China; ⊥ Laboratory of Atmospheric Chemistry, Paul Scherrer Institute, Villigen 5232, Switzerland; # EPFL, School of Architecture, Civil and Environmental Engineering, Sion 1951, Switzerland

**Keywords:** new particle formation, condensation sink, particles’ chemical composition, ammonium nitrate, urban environments

## Abstract

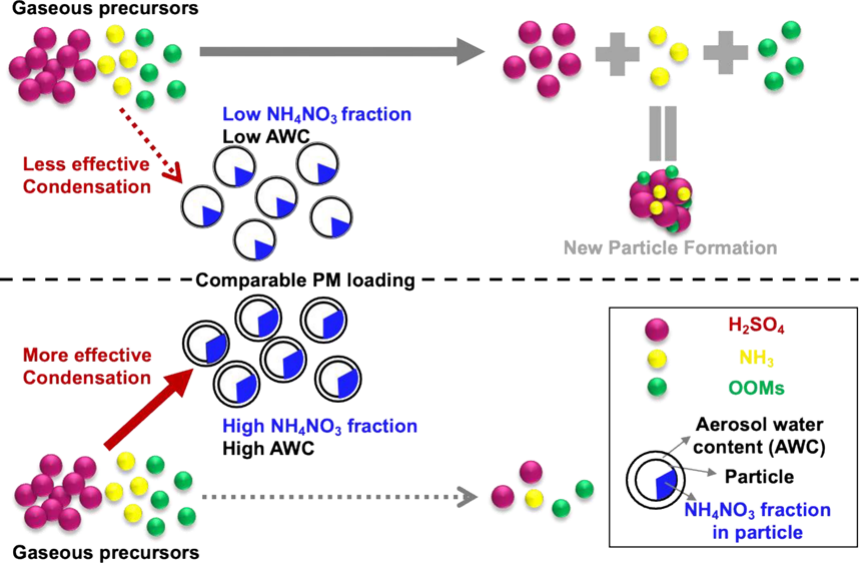

Relatively high concentrations of preexisting particles, acting as a condensation sink (CS) of gaseous precursors, have been thought to suppress the occurrence of new particle formation (NPF) in urban environments, yet NPF still occurs frequently. Here, we aim to understand the factors promoting and inhibiting NPF events in urban Beijing by combining one-year-long measurements of particle number size distributions and PM_2.5_ chemical composition. Our results show that indeed the CS is an important factor controlling the occurrence of NPF events, with its chemical composition affecting the efficiency of the background particles in removing gaseous H_2_SO_4_ (effectiveness of the CS) driving NPF. During our observation period, the CS was found to be more effective for ammonium nitrate-rich (NH_4_NO_3_-rich) fine particles. On non-NPF event days, particles acting as CS contained a larger fraction of NH_4_NO_3_ compared to NPF event days under comparable CS levels. In particular, in the CS range from 0.02 to 0.03 s^–1^, the nitrate fraction was 17% on NPF event days and 26% on non-NPF event days. Overall, our results highlight the importance of considering the chemical composition of preexisting particles when estimating the CS and their role in inhibiting NPF events, especially in urban environments.

## Introduction

Aerosol particles have significant effects on human health and climate.^1−4^ New particle formation (NPF), resulting from gas-to-particle conversion, has gained wide attention as it is one of the most important sources of atmospheric particles on a global scale.^5−9^ In the atmosphere, the major sink for gaseous precursors is their loss to the preexisting particle population, especially to particles in the accumulation mode size range.^10,11^ In practice, condensation sink (CS) describes how rapidly gaseous molecules are scavenged by preexisting aerosols.^12,13^

Previous studies have reported NPF events in both low-CS environments^6^ and rather polluted megacities in China and India.^14−23^ Although NPF is more likely to occur during clean episodes in these highly polluted megacities, the observed CS was still too high for NPF to occur according to theoretical calculations.^9,10,24^ A possible explanation could be that the scavenging of vapors responsible for the cluster formation and initial growth by available preexisting particles is less efficient than estimated.^10,24^ In general, the loss of a certain vapor could be affected by the characteristics of the preexisting particles. However, when the CS is calculated, traditionally, only the particle number size distribution (PNSD) is considered.^25^ This assumes that other factors such as the particles’ morphology, physical state, and chemical composition do not affect the CS. However, Tuovinen et al.^26^ showed theoretically that a larger contact angle leads to a possibly less efficient heterogeneous nucleation of a vapor consisting of sulfuric acid (H_2_SO_4_) and dimethylamine, resulting in a substantially reduced effective CS. Although the direct links between particle characteristics and the CS are still unknown, the contact angle of the heterogeneous nucleation, which is the initial step of condensation, is thought to strongly depend on the chemical composition of the preexisting particles.^27^

Thus, our aim is to identify the connection among the chemical composition of particles, CS effectiveness, and the occurrence of NPF events in Beijing, a polluted megacity. This is the first study that provides a plausible explanation for the influence of chemical composition on CS effectiveness using one-year-long urban environment ambient measurements.

## Materials and Methods

Here, we combined measurements of PNSDs, chemical composition of PM_2.5_, gaseous precursors, and meteorological parameters in urban Beijing, China, from March 1, 2018, to March 1, 2019 (Table S1). The sampling site is located on the west campus of Beijing University of Chemical Technology (39°56′31″ N, 116°17′50″ E). A scanning mobility particle sizer (SMPS, model 3936, TSI), a differential mobility particle sizer (DMPS, custom built), and a neutral cluster and air ion spectrometer (NAIS, model 4-11, Airel) were deployed to measure the PNSDs (Text S1).^28^ Black carbon (BC) was measured using a seven-wavelength aethalometer (AE33, Magee Scientific Corp.), and the nonrefractory chemical compositions of fine particles (NR-PM_2.5_), including organics (Org), sulfate (SO_4_), nitrate (NO_3_), ammonium (NH_4_), and chloride (Chl), were measured using a time-of-flight aerosol chemical speciation monitor (ToF-ACSM, Aerodyne Research Inc.)^29^ (Text S2). Diffusion dryers were set up in front of these instruments to ensure dry sampling of the aerosol particles. Measured PM_2.5_ (NR-PM_2.5_ + BC) agreed well with mass concentrations converted from PNSDs, indicating that those instruments performed steadily and in parallel (Text S3).

In addition, a chemical ionization atmospheric pressure interface time-of-flight mass spectrometer (CI-APi-ToF, Aerodyne Research, Inc.) was used to measure H_2_SO_4_ (H_2_SO_4meas_) from December 26, 2018, to March 1, 2019 (Text S4).^30^ Alkenes were measured using a single-photon ionization time-of-flight mass spectrometer (SPI-MS 3000R, Hexin Mass Spectrometry) (Text S5). The concentrations of sulfur dioxide (SO_2_) and ozone (O_3_) were also monitored (model 43i and model 49i, respectively, Thermo). The global radiation (GlobRad) intensity was measured at the rooftop of the five-floor building using a CMP11 pyranometer.^31^ Detailed information about the sampling site and instruments’ operation is available in previous studies.^11,18,32,33^

Chemical-dependent hygroscopicity parameters (κ) were estimated following a simple mixing rule combining hygroscopicity of the individual dry particles’ components with their particle volume fraction (Text S6).^34^ Although the calculation of κ had uncertainties by assuming a constant κ for organics,^35^ our sensitivity tests suggested that this minor difference in κ caused by the secondary organic aerosols (SOA) can be neglected during our observation period (Texts S2 and S6). To calculate the mass concentration of the aerosol water content (AWC), a thermodynamic model, ISORROPIA version 2.1, based on inorganic aerosol components was used (Text S6).^36^

The CS is calculated from the PNSD as^25^
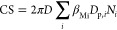
1where *D* is the diffusion coefficient of the condensing vapor, in our case H_2_SO_4_.^37,38^*D*_p_ and *N* are the particle diameter and its corresponding number concentration, respectively. *β*_M_ is the transitional regime correction factor.

NPF events were classified on the basis of the PNSD in which the appearance of fresh particles in the cluster and nucleation mode size ranges (<25 nm) showing signs of growth independent of the meteorological conditions during that day (Text S7).^32,39,40^

In addition, we estimated H_2_SO_4_ concentrations (H_2_SO_4proxy_) derived for Beijing by Dada et al.^11^ (Text S8). By assuming a steady state between H_2_SO_4_ production and loss, we can estimate H_2_SO_4proxy_:
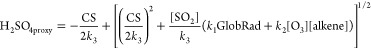
2where *k*_1_–*k*_3_ represent the coefficient of H_2_SO_4_ production term associated with daytime SO_2_–OH reaction, the coefficient of H_2_SO_4_ production via stabilized Criegee intermediates produced by the ozonolysis of alkenes mostly during nighttime, and the clustering coefficient, respectively. In this study, we used the values for *k*_1_–*k*_3_ previously determined for Beijing (2.0 × 10^–8^, 1.5 × 10^–29^, and 7.0 × 10^–9^, respectively).^11^

## Results and Discussion

### CS and New Particle Formation Events

**Figure 1:**
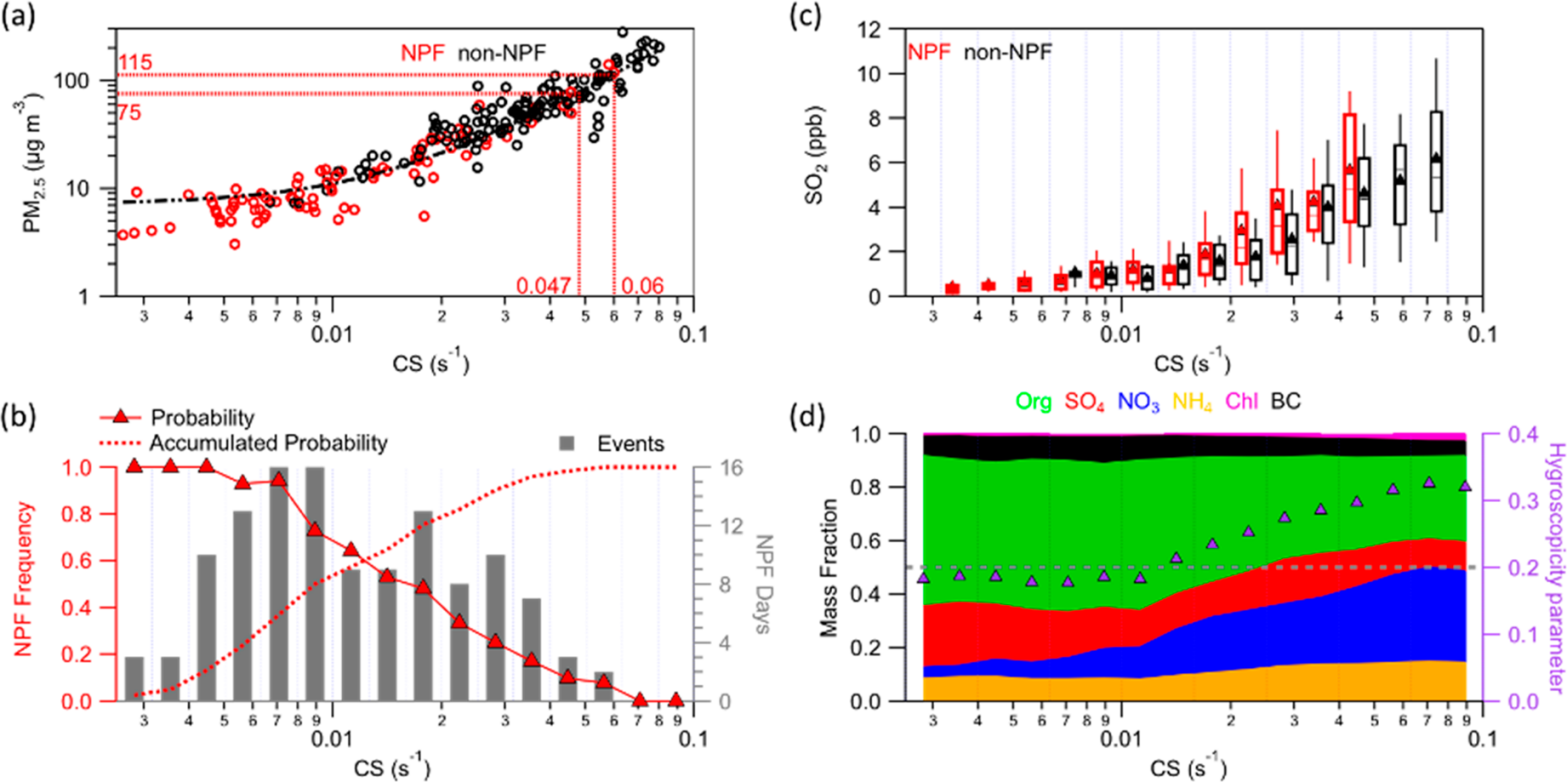
Atmospheric aerosols, gaseous precursors, and the occurrence of new particle formation events. (a) Daytime average (from 10:00 to 15:00) PM_2.5_ concentration vs daytime average (from 10:00 to 15:00) condensation sink (CS). (b) Distribution of NPF event probability (red triangles, left axis) and accumulated NPF probability (dotted line, left axis), and the number of NPF event days (gray bars, right axis) as a function of daytime average (from 10:00 to 15:00) condensation sink (CS). (c) Concentration of SO_2_ as a function of CS during NPF (red) and non-NPF (black) days. Only daytime data (from 10:00 to 15:00) are considered. Within each box, which corresponds to a logarithmic CS bin, the median (middle horizontal line), mean (solid triangles), 25th and 75th percentiles (bottom and top ends of the box, respectively), and 10th and 90th percentiles (bottom and top whiskers, respectively) are shown. (d) Fraction of particle chemical composition and hygroscopicity parameter (triangle) as a function of condensation sink (CS). The gray dashed line refers to the 50% mass fraction.

During our one -year observation period, the hourly averaged PM_2.5_ varied dramatically from 1 to 445 μg m^–3^ with a median concentration of 33 μg m^–3^ (Figure S2). Consistently, the hourly averaged CS varied from 0.0011 to 0.14 s^–1^ with a median value of 0.026 s^–1^. As expected, the CS was high concurrent with a high PM_2.5_ (Figure 1a). Although the high CS levels in Beijing were traditionally thought to suppress NPF, NPF events were observed on 122 of 306 days (40%), ranking Beijing among locations with the highest NPF frequency around the world.^10,41^ NPF in Beijing occurred during a daytime (from 10:00 to 15:00) average CS (CS_daytime-avg_) between 0.002 and 0.06 s^–1^ with >98% of the events occurring when the CS_daytime-avg_ was <0.047 s^–1^ (corresponding to PM_2.5_ < 75 μg m^–3^). The variability in CS_daytime-avg_ was an important factor determining the occurrence of NPF events. When CS_daytime-avg_ was <0.008 s^–1^, the probability of NPF was >90%. The probability of NPF gradually decreased with an increase in CS_daytime-avg_, to become 0% when CS_daytime-avg_ was >0.06 s^–1^ (Figure 1b). Thus, a CS of ∼0.06 s^–1^ appears to be the threshold for the NPF to occur in Beijing within our observation period. Fifty percent of the NPF events were observed when CS_daytime-avg_ was >0.01 s^–1^ in Beijing, and even 20% of the NPF events occurred when CS_daytime-avg_ was >0.02 s^–1^.

The occurrence of NPF depends on the competition between vapor sources and their sinks.^42^ As shown in Figure 1c, the SO_2_ concentration increased with CS_daytime-avg_, which is explained by similar sources of accumulation mode particles and SO_2_ : regional transport.^32^ A high level of SO_2_ has the potential to produce a higher level of H_2_SO_4_, which is the most important gaseous precursor driving the nucleation process.^18,19,37^ However, a high CS (CS_daytime-avg_ > 0.06 s^–1^) together with a low level of radiation (UVB < 0.3 W m^–2^) resulted in a decrease in H_2_SO_4meas_, shutting off NPF completely (Figures S5 and S6). Interestingly, both NPF event and non-NPF event days were observed under the same CS_daytime-avg_ levels in the range of 0.01–0.06 s^–1^, with varying NPF frequencies (from 60% to 10%). Within this moderate CS_daytime-avg_, although non-NPF event days showed lower levels of UVB associated with low levels of solar radiation in winter, H_2_SO_4meas_ was only slightly lower than or comparable to H_2_SO_4meas_ on NPF event days. A comparable H_2_SO_4_ under lower UVB levels can be explained by the formation of H_2_SO_4_ from alkene and ozone reactions and the associated stabilized Criegee intermediate pathway, an important formation pathway in the winter in Beijing.^11^ Hence, H_2_SO_4_ can behave differently under similar CSs, resulting in NPF event and non-NPF event days.

### Influence of the Chemical Composition on CS Effectiveness

In Beijing, the chemical composition of aerosol particles varied among different CSs, although all components increased with CS (Figure 1d and Figure S7). When the CS was <0.01 s^–1^, the fraction of organic and inorganic aerosols remained relatively stable: the organic fraction typically constituted 50–60% of the total particle mass. However, when the CS was >0.01 s^–1^, the mass fraction of secondary inorganic aerosols (SIA) increased significantly from ∼34% (CS = 0.01 s^–1^) to ∼61% (CS = 0.08 s^–1^) (Figure 1d). Accounting for ≤35% of the total mass, NO_3_ was the main contributor to the increasing SIA when the CS was >0.01 s^–1^, while the fraction of SO_4_ remained relatively stable. Such drastic changes in chemical compositions can affect the properties of the particles, e.g., hygroscopicity.^34^ While the particles’ hygroscopicity remained low at CSs of <0.01 s^–1^ (κ < 0.2), the particles became substantially more hygroscopic (κ increased from 0.18 to 0.33) with an increase in CS (Figure 1d). The increased hygroscopicity is driven by the increased ammonium nitrate (NH_4_NO_3_) fraction at an enhanced CS (Figure S8a). As a consequence, particles are more likely to absorb water and swell to larger sizes,^43,44^ which can further promote the uptake of gaseous vapors altering the effectiveness of CS (Text S9 and Figure S8). These observations suggest that the particles’ chemical composition affects the efficiency of the CS for both condensable and reactive uptake of vapor molecules.

**Figure 2:**
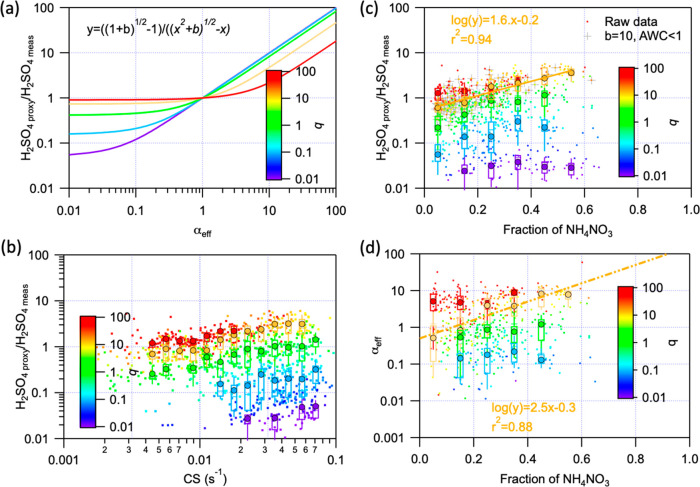
Effectiveness of a condensation sink (CS) under varying conditions. (a) Evolution of the ratio between the estimated H_2_SO_4_ (H_2_SO_4proxy_) and measured H_2_SO_4_ (H_2_SO_4meas_) concentration as a function of parameter *α*_eff_ in theory. (b and c) Evolution of the ratio between the estimated H_2_SO_4_ (H_2_SO_4proxy_) and measured H_2_SO_4_ (H_2_SO_4meas_) concentration as a function of the CS and mass fraction of ammonium nitrate during our observation, respectively. (d) Connection between parameter *α*_eff_ and the fraction of ammonium nitrate. *α*_eff_, indicating the efficiency of the particles in removing vapors, is estimated from eq 6 by comparing H_2_SO_4proxy_ to H_2_SO_4meas_. The color changed from purple to red with *b* in eq 5 increasing from 0.01 to 100. Within each box, the mean (middle horizontal line), median (filled circles), 25th and 75th percentiles (bottom and top ends of the box, respectively), and 10th and 90th percentiles (bottom and top whiskers, respectively) are shown. Values with *b* ranging from 10^0.5^ to 10^1.5^ and an aerosol water content (AWC) of <1 are marked with crosses in panel c. The yellow lines in panels c and d are linear fittings of values with *b* ranging from 10^0.5^ to 10^1.5^.

To confirm this hypothesis and given that there is no direct way to measure the effectiveness of the CS for H_2_SO_4_ in the atmosphere, we estimate CS effectiveness by comparing H_2_SO_4proxy_ to H_2_SO_4meas_. The ratio between H_2_SO_4proxy_ and H_2_SO_4meas_ (H_2_SO_4proxy_/H_2_SO_4meas_) during the daytime is a function of CS (Figure S9), which suggests that the effectiveness of the CS increases with the calculated CS. This observation implies that accounting for the effectiveness of CS by introducing an additional coefficient (*α*_eff_) is needed when estimating H_2_SO_4proxy_. Thus, H_2_SO_4proxy_/H_2_SO_4meas_ can be expressed as (based on eq 2)
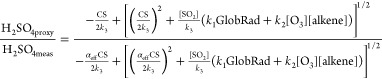
3Equation 3 can be simplified to
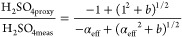
4where *b* is
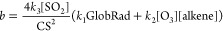
5Further, *α*_eff_ can be calculated from eq 6:
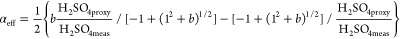
6The connection between factor *α*_eff_ and H_2_SO_4proxy_/H_2_SO_4meas_ is nonlinear but depends on the relative magnitudes of the H_2_SO_4_ sources and sinks in its budget equation (summarized in eq 5 as *b*). In theory, the CS is more effective at a higher H_2_SO_4proxy_/H_2_SO_4meas_ under the condition that *b* remains constant as illustrated by Figure 2a. It should be noted that *α*_eff_, indicating the efficiency of the particles in removing vapors, is not the simple effectiveness of the CS, which represents the sticking probability, always <1.^10,26^ Because the CS was calculated from the dry PNSD rather than the wet PNSD in the atmosphere and *α*_eff_ was estimated from H_2_SO_4proxy_/H_2_SO_4meas_, *α*_eff_ could be >1 (Text S10).

Figure 2b shows the observed dependency of H_2_SO_4proxy_/H_2_SO_4meas_ on CS. The H_2_SO_4proxy_/H_2_SO_4meas_ and CS seem to be not correlated overall, while positive correlations were observed within constant *b* values. Considering a *b* value of 10 as a typical value during the whole period, when the CS was <0.01, H_2_SO_4proxy_/H_2_SO_4meas_ was relatively stable (median value of ∼0.7) but increased to ∼4.0 when the CS increased to 0.06 s^–1^. In particular, this variation in H_2_SO_4proxy_/H_2_SO_4meas_ appears to be associated with an increase in the NH_4_NO_3_ mass fraction, and a high positive correlation between log(H_2_SO_4proxy_/H_2_SO_4meas_) and the NH_4_NO_3_ fraction was observed [*r*^2^ = 0.9 (Figure 2c)]. Relying on H_2_SO_4proxy_/H_2_SO_4meas_, we could obtain the relationship between *α*_eff_ and the NH_4_NO_3_ fraction (Figure 2d). Although the exact relationship depends on the varying *b*, all of them indicated that the effectiveness of CS increased with an increase in the NH_4_NO_3_ fraction. Here, we need to state that although other chemical compounds, e.g., SO_4_ and SOA, could also affect CS effectiveness, NH_4_NO_3_ plays a more dominant role in defining CS effectiveness during our observation period (Figure S10).

An increase in the NH_4_NO_3_ fraction results in a higher κ and hence an increased AWC. However, while H_2_SO_4proxy_/H_2_SO_4meas_ overall increases at an enhanced AWC/PM_2.5_ ratio, the increase in hygroscopicity and AWC in the particles does not fully explain the increase in CS effectiveness (Figure S11). Under very low AWC (<1 μg m^–3^) and low-RH (<20%) conditions, the relation between H_2_SO_4proxy_/H_2_SO_4meas_ and the NH_4_NO_3_ fraction remained consistent at a *b* value of 10 (Figure 2 and Figure S11). This implies that additional characteristics related to NH_4_NO_3_ (other than hygroscopicity) allow particles with a high NH_4_NO_3_ mass fraction to act as a more efficient sink for gaseous H_2_SO_4_. A possible explanation is that the surface of particles could become sticky due to a higher fraction of NH_4_NO_3_, which is a metastable liquid in the atmosphere.^45,46^

### Influence of Chemical Composition and CS Effectiveness on NPF Occurrence

**Figure 3:**
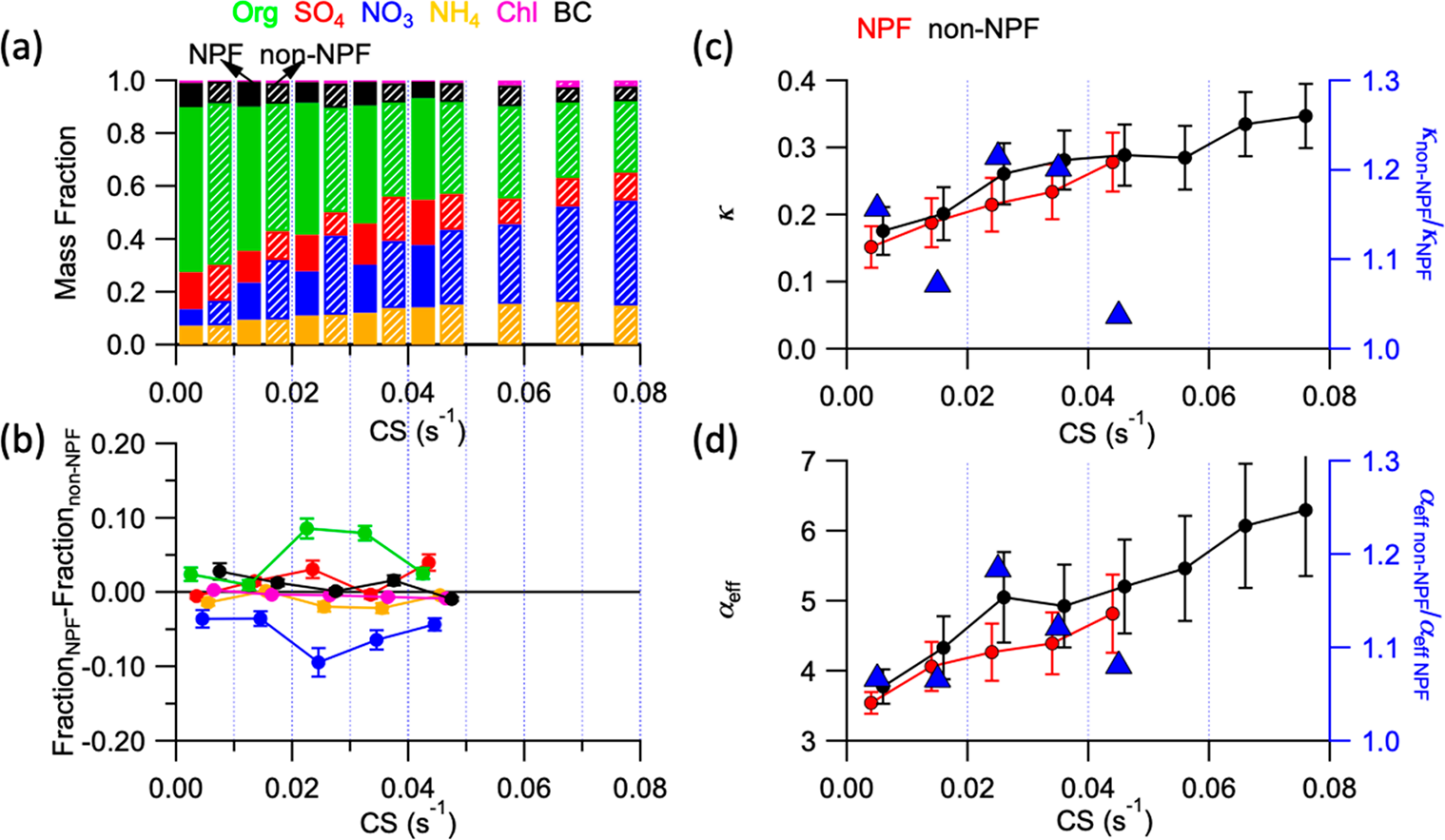
Link among particles’ chemical composition, CS effectiveness, and the impact on NPF. (a) Chemical composition of preexisting particles during the daytime (from 10:00 to 15:00) and (b) difference in the chemical mass fraction of particles between NPF and non-NPF days under different condensation sinks (CSs) during the daytime (from 10:00 to 15:00). (c) Evolution of hygroscopicity (κ) on NPF and non-NPF days (left axis) and ratio of hygroscopicity parameter between non-NPF and NPF days (κ_non-NPF_/κ_NPF_, right axis) as a function of CS during the daytime (from 10:00 to 15:00). (d) Evolution of *α*_eff_ in NPF and non-NPF days (left axis) and ratio of parameter *α*_eff_ between non-NPF and NPF days (*α*_eff__non-NPF_/*α*_eff__NPF_, right axis) as a function of CS during the daytime (from 10:00 to 15:00).

Because H_2_SO_4_ is the main driver of NPF in Beijing,^18^ the varying effectiveness of particles acting as CS for H_2_SO_4_ could be the determining factor for the occurrence of NPF events. When comparing NPF event and non-NPF event days within a comparable CS_daytime-avg_ range, we found that the nitrate mass fraction was generally higher on non-NPF event days than on NPF event days (Figure 3a,b). This difference in the nitrate fraction was particularly large in the CS_daytime-avg_ range from 0.02 to 0.03 s^–1^, in which the nitrate fraction was 17% on NPF event days and 26% on non-NPF event days. Driven by the higher nitrate fraction, κ was higher during non-NPF event days for the same CS_daytime-avg_ (Figure 3c), suggesting particles on non-NPF event days are more effective in taking up water vapors. Consistently, assuming that log(*α*_eff_) is a linear function of the NH_4_NO_3_ fraction during the daytime (Figure S9c), we found *α*_eff_ was 7–18% higher during non-NPF event days than during NPF event days, and thus more effective removal of gaseous H_2_SO_4_ needed for NPF (Figure 3d). Although an AWC is not the driver behind the effectiveness of a CS, a higher AWC associated with a higher κ and a higher RH could also play a role in affecting CS effectiveness by providing a larger surface area and promoting heterogeneous reactions (Text S9). In summary, the NH_4_NO_3_ fraction in the background particles controls CS effectiveness, which in turn influences NPF in Beijing.

## Supporting Information Available

Detailed sampling information and calculation methods that were applied in this study (Texts S1–S9), information about the instruments (Table S1), calculations of densities and hygroscopicity parameters used in this study (Table S2), parallel comparison of particle number size distribution measurements (Figure S1), time series of the condensation sink and particle mass (Figure S2), comparison between secondary organic aerosol concentrations from different methods (Figure S3), sensitive tests of hygroscopicity parameters (Figure S4), global radiation as a function of condensation sink (Figure S5), measured sulfuric acid as a function of condensation sink (Figure S6), chemical compositions of PM_2.5_ as a function of condensation sink (Figure S7), variations of aerosol water content, sulfur oxidation ratios, and condensation sink (Figure S8), effectiveness of the condensation sink during the daytime (Figure S9), influence of chemical compositions on condensation sink (Figure S10), aerosol water content and effectiveness of the condensation sink (Figure S11), and relative humidity, aerosol water content, and sulfur oxidation ratios as a function of condensation sink (Figure S12) (PDF)
