# Supplementary material for: Influence of Aerosol Chemical Composition on Condensation Sink Efficiency and New Particle Formation in Beijing
Source: Environ Sci Technol Lett. 2022 Apr 12;9(5):375–82. doi: 10.1021/acs.estlett.2c00159 (PMC9097482; doi:10.1021/acs.estlett.2c00159)
Supplement: Supplementary file 1 — ez2c00159_si_001.pdf [file ez2c00159_si_001.pdf]

Supplementary information for "The influence of aerosol chemical composition on condensation sink efficiency and new particle formation in Beijing"

Wei Du[1,2,#], Jing Cai[2,#], Feixue Zheng[1], Chao Yan[2], Ying Zhou[1], Yishuo Guo[1], Biwu Chu[3] , Lei Yao[2], Liine M. Heikkinen[2], Xiaolong Fan[1], Yonghong Wang[2], Runlong Cai[2], Simo Hakala[2], Tommy Chan[2], Jenni Kontkanen[2], Santeri Tuovinen[2], Tuukka Petäjä[2], Juha Kangasluoma[2], Federico Bianchi[2], Pauli Paasonen[2], Yele Sun[4], Veli-Matti Kerminen[2], Yongchun Liu[1], Kaspar R. Daellenbach[1,2,5,*], Lubna Dada[2,5,6,*], Markku Kulmala[1,2]

Affiliations:

[1] Aerosol and Haze Laboratory, Beijing Advanced Innovation Center for Soft Matter Science and Engineering, Beijing University of Chemical Technology, Beijing, China

[2] Institute for Atmospheric and Earth System Research / Physics, Faculty of Science, University of Helsinki, Finland

[3] State Key Joint Laboratory of Environment Simulation and Pollution Control, Research Center for Eco-Environmental Sciences, Chinese Academy of Sciences, Beijing, 100085, China

[4] State Key Laboratory of Atmospheric Boundary Layer Physics and Atmospheric Chemistry, Institute of Atmospheric Physics, Chinese Academy of Sciences, Beijing 100029, China

[5] Laboratory of Atmospheric Chemistry, Paul Scherrer Institute, Villigen, 5232, Switzerland

[6] EPFL, School of Architecture, Civil and Environmental Engineering, Sion, 1951, Switzerland

[#] These authors contributed equally.

Corresponding authors:

Lubna Dada (lubna.dada@helsinki.fi),

Kaspar R. Daellenbach (kaspar.dallenbach@helsinki.fi).

Text S1. Comparison between SMPS and DMPS

A Scanning Mobility Particle Sizer (SMPS, Model 3936, TSI) and a Differential Mobility Particle Sizer (DMPS, Custom built) were deployed to measure the PNSDs in the size ranges from 14 to 737 nm and 6 to 840 nm, respectively (Fig. S1, Text S1). In addition, a neutral cluster and air ion spectrometer (NAIS, model 4-11, Airel, Estonia) measured the number size distributions of total particles (2.5–42nm) and ions (0.7– 42nm). The data from March 1st, 2018 to May 30th, 2018 were measured by the SMPS and from June 1st, 2018 to March 1st, 2019 by the DMPS. In order to ensure consistency during our continuous measurements, the particle number size distributions (PNSDs) from the two instruments were compared for the following period when they were simultaneously measuring from June 11th, 2018 to June 30th, 2018 (Fig. S1). The comparison results show that the average PNSDs matched well (r^2=0.97. Further, by assuming that the real size distribution during the overlapping measuring period is the average of the size distributions measured by both instruments ($PNSD_{(SMPS+DMPS)/2}$), a scaling factor method was developed to correct the systematic difference between the two instruments. Thus, the size-segregated ratios of $PNSD_{SMPS}$ (or $PNSD_{DMPS}$) to $PNSD_{(SMPS+DMPS)/2}$ during the overlapping measuring period were applied as the size-resolved correction coefficients to calibrate SMPS (or DMPS), respectively.

Text S2. Measurement of particle chemical compositions

Black carbon (BC) in $PM_{2.5}$ was measured using a seven-wavelength Aethalometer (AE33, Magee Scientific Crop.). The non-refractory chemical compositions of fine particles (NR-$PM_{2.5}$), including organics (Org), sulfate (SO_4), nitrate (NO_3), ammonium (NH_4), and chloride (Chl), were measured using an online Time-of-Flight Aerosol Chemical Speciation Monitor (ToF-ACSM, Aerodyne Research Inc. U.S.) equipped with a $PM_{2.5}$ aerodynamic lens and a standard vaporizer. Tof-ACSM was frequently calibrated (once in ~1 month) with NH_4NO_3 for ionization efficiency (IE) and with pure standards of NH_4Cl, $(NH_4)_2SO_4$ for relative ionization efficiency (RIE). During the whole campaign period, stable IE/air beam signals and RIE were achieved (±20% for standard/average IE/air beam), suggesting the stable performance of Tof-ACSM. The average values of RIEs (for sulfate, nitrate, ammonium, and chloride: were 0.86, 1.05, 4.0, and 1.5, respectively). The IE/air beam averaged from all calibrations were used to calculate the concentrations of NR-$PM_{2.5}$ chemical components. We also excluded positive bias on organic CO_2^+ due to high levels of NO_3. A more detailed description of the Tof-ACSM settings and artefacts' corrections can be found in Cai et al. [2].

To resolve the SOA component from the Tof-ACSM measurement, two source apportionment methods were applied

1) m/z44 tracer method according to empirical equations from Ng et al. [3]:

$$SOA = a \times C_{44} + b \qquad (1)$$

Where C_{44} is the equivalent mass concentration of m/z44 (a tracer ion of SOA).

2) The Positive Matrix Factorization (PMF) method:

We solved the PMF by the multilinear engine (ME-2) algorithm implemented within the toolkit SoFi, Source Finder. The detailed information on the PMF method was given in Kulmala et al. [4].

Overall, SOA resolved from the tracer method and that from the PMF method correlated well (r^2=0.95, Fig. R8, added as Fig. S3 in our revised manuscript), and SOA from the tracer method was used in the further calculation in this study.

Text S3. Comparison between measured by ACSM and converted mass from PNSDs

Since the particle chemical composition and size measurements are based on different detection methods, two kinds of diameter definitions were used in parallel throughout this study. The diameter range of the SMPS and DMPS measurements was defined by the electrical mobility diameter (D_m)

and the diameter range of the ACSM measurements was defined by the vacuum aerodynamic diameter (D_{va}). With the assumption of spherical particles, D_{va} approximately equals D_m multiplied by average particle density ($\rho_{particle\_avg}$ = 1.5 g cm^{-3} in this study). Although the upper cut-off size of the SMPS and DMPS was still smaller than that of the ACSM and the AE33, those instruments were typically considered to be comparable since in most cases $PM_{2.5}$ concentrations were dominated by PM_1 in Beijing (Table S1) [5, 6]. This is also consistent with our dataset comparison between mass concentration converted from PNSD and measured $PM_{2.5}$ (NR-$PM_{2.5}$ + BC). To convert the particle number size distributions to mass concentration, apart from spherical particle assumption, time varied particle densities by using their measured chemical composition were also applied [7]. The derived time series of the calculated mass concentration from PNSD compared well to $PM_{2.5}$ measured by ACSM and AE33 (r^2 = 0.88, slope=0.95, Fig. S3), indicating that submicron aerosols dominated the mass of $PM_{2.5}$ even though possible uncertainties may be created in the particle density calculation and the ignorance of dust and metal elements, which could not be measured by ACSM. In general, the good correlation and slope close to 1 indicated that those instruments performed steadily and in parallel in terms of chemical mass, number, and size distributions of the particles during the measurement period.

Text S4 Measurement of Sulfuric acid (H_2SO_4)

H_2SO_4 are measured by a Chemical Ionization Atmospheric Pressure interface Time-of-Flight mass spectrometer (CI-APi-TOF, Aerodyne Research, Inc.) equipped with a nitrate chemical ionization source [8]. The signal of H_2SO_4 is calibrated with known concentrations of gaseous sulfuric acid produced by the reaction of SO_2 and OH radicals that are formed by UV photolysis of water vapor, similar to the protocols in previous literature [9]. We obtained a calibration coefficient of 6.07×10^9 molecule cm^{-3} for our instrument. Detailed information on the instrument and its setting, calibrations, and corrections can be found in Yan et al. [10].

Text S5 Measurement of Alkenes

VOCs were measured using a single-photon ionization time-of-flight mass spectrometer (SPI-MS 3000R, Hexin Mass Spectrometry) with unit mass resolution (UMR). The detailed information on the instrument operation could be found in Liu et al [11] and Wang et al [12]. 0.8 L min^{-1} of filtered air was sucked from the whole sampling tube and heated to 80 ºC in the inlet. VOCs were selectively enriched continuously through a polydimethylsiloxane (PDMS) membrane and then ionized by VUV light (10.5 eV) with a deuterium lamp. The concentration of VOCs was determined with the time-of-flight mass spectrometer (ToF-MS) based upon external standard curves of PAMS and TO-15 standard gases (Linde Electronics & Specialty Gases, USA). Calibration was performed every week. The alkenes included here are propylene, butylene, butadiene, isoprene, pentene and hexene.

Text S6. Hygroscopicity parameter and Aerosol water content

For a given internal mixture, the hygroscopicity parameter (κ) can be predicted by a simple mixing rule on the basis of chemical volume fractions [13]:

$$\kappa = \sum_i \varepsilon_i \kappa_i \qquad (2)$$

Here, κ_i and ε_i are the hygroscopicity parameters and volume fraction for component *i* (dry) in the mixture. We derived ε_i from the particle chemical composition measured by ACSM. Table S2 gave the densities and hygroscopicity parameters for chemical compounds used in this study. The ion-pairing scheme presented by Gysel et al.[14] was used to convert the ion mass concentrations to the mass concentrations of their corresponding inorganic salts. The volume fraction of each species was then calculated from the particle mass concentration divided by its density. Unlike inorganic salts, the hygroscopicity of organic aerosols is not well recognized. Primary organic aerosols (POA) are the unoxygenated component and are generally treated as hydrophobic material with κ_{POA} = 0. κ of

laboratory-generated secondary organic aerosols (SOA) varied from 0.01 to 0.2, while it was assumed to be 0.1 in many studies. [15] Further, κ for organic aerosols can be calculated. Wu et al. [16] found that κ_{OA} for organic aerosols is approximately 0.06 in Beijing, which was used in our study. We further tested the influence of SOA on κ. In the two sensitive tests (Table S2), we calculated the time series of κ_{test1} and κ_{test2} during our observation by assuming that SOA has κ_{SOA} value of 0.05 and 0.15, respectively. As shown in Fig. S4, the calculated κ_{test1} and κ_{test2} were similar to κ using κ_{OA} value of 0.06 for organic aerosols.

ISORROPIA model has been extensively applied to predict aerosol water content (AWC) with the input of ambient temperature, relative humidity (RH), as well as the concentrations of chemical components of fine particulate matters [17, 18]. In our analysis, the mass concentrations of NH_4, SO_4, Chl, NO_3 were measured by ACSM as well as meteorological conditions measured by a Vaisala Weather station data acquisition system (AWS310, PWD22, CL51, Metcon) were used as input. Although there are uncertainties on AWC calculation when not considering the influence of Na, Ca, K, and Mg, the uncertainty remains minimal due to the insignificant mass contribution of crustal ions to $PM_{2.5}$ in Beijing [6, 19].

Text S7. Classification of new particle formation (NPF) events

The original method has been introduced by Dal Maso, et al. [20]. However, for this study, the method was modified to fit the criteria in megacities, where the background particle number concentration is rather high and to include observations from sub-3 nm clusters [21-23]. The criteria adopted here include: if a burst of sub-3 nm particles is followed by further particle growth reaching diameters exceeding at least 25 nm, the day is classified as an NPF day. When neither a burst of sub-3 nm particles nor the subsequent growth of newly formed particles is observed, the day is classified into a non-NPF day. Within the 306 days of observations, 122 NPF events were identified.

Text S8. Sulfuric acid proxy

We used a recent proxy for H_2SO_4 derived by Dada et al.[24] based on the physical understanding of the sources and sinks of H_2SO_4:

$$\frac{d[H_2SO_4]}{dt} = k_1 GlobRad[SO_2] + k_2[O_3][Alkene][SO_2] - CS[H_2SO_4] - k_3[H_2SO_4]^2 \qquad (3)$$

Here, k_1 represents the coefficient of H_2SO_4 production term due to daytime SO_2-OH reaction, k_2 is the coefficient of H_2SO_4 production via stabilized Criegee Intermediates produced by the ozonolysis of alkenes mostly during nighttime. The third term in Equation 2 represents the loss of H_2SO_4 to CS. The fourth term considers the additional loss of H_2SO_4 to cluster formation, and k_3 represents the clustering coefficient.

Text S9. Total sulfur and sulfur oxidation ratio

To characterize the fraction of sulfur that exist in the particle phase, the sulfur oxidation ratio (SOR) was calculated as the molar ratio of the gas phase (n_{SO_2}) to the sum of gas-phase (n_{SO_2}) and particle phase (n_{SO_4}) according to equation (4):

$$SOR = n_{SO_4}/(n_{SO_4} + n_{SO_2}) \qquad (4)$$

Within a similar CS range, the SOR was substantially higher at elevated AWC, suggesting a more efficient conversion of SO_2 to particle-phase sulphate, leaving less gaseous sulfur available for nucleation (Fig. S8). In addition, more gaseous sulfur was transformed to particle-phase on non-NPF days, indicated by the higher sulfur oxidation ratio (Fig. S12).

Text S10. Wet CS, the effectiveness of CS, and parameter α_{eff}

To calculate wet CS (CS_{wet}), we estimated the diameter of wet particles (d_{wet}) [25, 26]:

$$d_{wet} = GFD_p \qquad (5)$$

Where D_p is the measured dry particle diameter. And GF is the hygroscopicity growth factor calculated as follows:

$$GF = \left[1 + \kappa\left(\frac{a_w}{1-a_w}\right)\right]^{1/3} \qquad (6)$$

Where κ is the size-dependent hygroscopicity parameter, and a_w is water activity obtained by:

$$a_w = \frac{RH}{exp\left(\frac{4\sigma M_W}{RTD_p\rho_W}\right)} \qquad (7)$$

Where σ is the droplet surface tension, M_w is the molecular weight of water, ρ_w is the density of liquid water, R is the universal gas constant, and T is the temperature. The size-dependent κ, which were assumed to be 0.24, 0.25, 0.28, 0.3, 0.35 for 30, 50, 100, 200, and >250 nm particles based on the measurements in Hyytiälä, a forest station where the hygroscopicity of particles was widely studied [27]. This assumption is comparable to that observed in China[25, 28].

Effectiveness of CS is defined as the ratio between the real CS ($CS_{effective}$) and observed CS ($CS_{observed}$) in Kulmala et al. [29]. $CS_{effective}$ is either the same or less than $CS_{observed}$ since it is still unclear whether all molecular clusters that collide with pre-existing particles also stick with them. Thus, the effectiveness of CS could not be higher than 1. Effectiveness of CS less than 1 indicated that pre-existing particles are not effective as expected in removing gaseous precursors.

α_{eff} resolved from the modeled and measured sulfuric acid could be used to indicate the particles' efficiency in removing vapors. However, α_{eff} is a relative value but not the simple effectiveness of CS. The parameter α_{eff} in our study can be also written as the following expression when wet particles are considered.

$$\alpha_{eff} = \alpha'_{eff} \times CS_{wet}/CS \qquad (8)$$

Where α'_{eff} is the remaining indicator of the effectiveness of CS after wet CS (CS_{wet}) conditions are considered. Since CS was calculated by dry particle number size distributions, which is not the case in the real atmosphere, and α_{eff} was estimated from the modeled and measured sulfuric acid, α_{eff} could be higher than 1.

Table S1. Instruments information

Instruments	Size range (D_{va})*	Measurement	Resolution	Time period
Gas pollutant analyzers	--	SO_2, O_3	1 min	2018/3/1 – 2019/3/1
CI-APi-ToF	--	H_2SO_4	5 s	2018/12/26 – 2019/3/1
SPI-MS 3000R	--	Alkenes	3 min	2018/12/26 – 2019/3/1
ToF-ACSM	~60 – 2500 nm	Non-refractory chemical composition	5 min	2018/3/1 – 2019/3/1
AE33	<2500 nm	Black carbon	5 min	2018/3/1 – 2019/3/1
SMPS	~20 – 1100 nm	Particle number size distribution	5 min	2018/3/1 – 2018/7/1
DMPS	~10 – 1200 nm		5 min	2018/5/30 – 2019/3/1

*we assume that ambient aerosols are spherical particles and particle density is 1.5 g cm^{-3}.

Table S2. Densities (ρ) and hygroscopicity parameters (κ) for chemical compounds used in our study and sensitive tests.

<table>
<tr><th rowspan="2">Species</th><th rowspan="2">NH_4NO_3</th><th rowspan="2">NH_4HSO_4</th><th rowspan="2">$(NH_4)_2SO_4$</th><th colspan="2">Org</th><th rowspan="2">BC</th></tr>
<tr><th>POA</th><th>SOA</th></tr>
<tr><td rowspan="2">ρ (g cm^{-3})</td><td rowspan="2">1.72</td><td rowspan="2">1.78</td><td rowspan="2">1.77</td><td colspan="2">1.2</td><td rowspan="2">1.7</td></tr>
<tr><td>1.0</td><td>1.4</td></tr>
<tr><td>Our study (κ)</td><td>0.58</td><td>0.56</td><td>0.48</td><td colspan="2">0.06</td><td>0</td></tr>
<tr><td>Sensitive test 1 (κ_{test1})</td><td>0.58</td><td>0.56</td><td>0.48</td><td>0</td><td>0.15</td><td>0</td></tr>
<tr><td>Sensitive test 2 (κ_{test2})</td><td>0.58</td><td>0.56</td><td>0.48</td><td>0</td><td>0.05</td><td>0</td></tr>
</table>

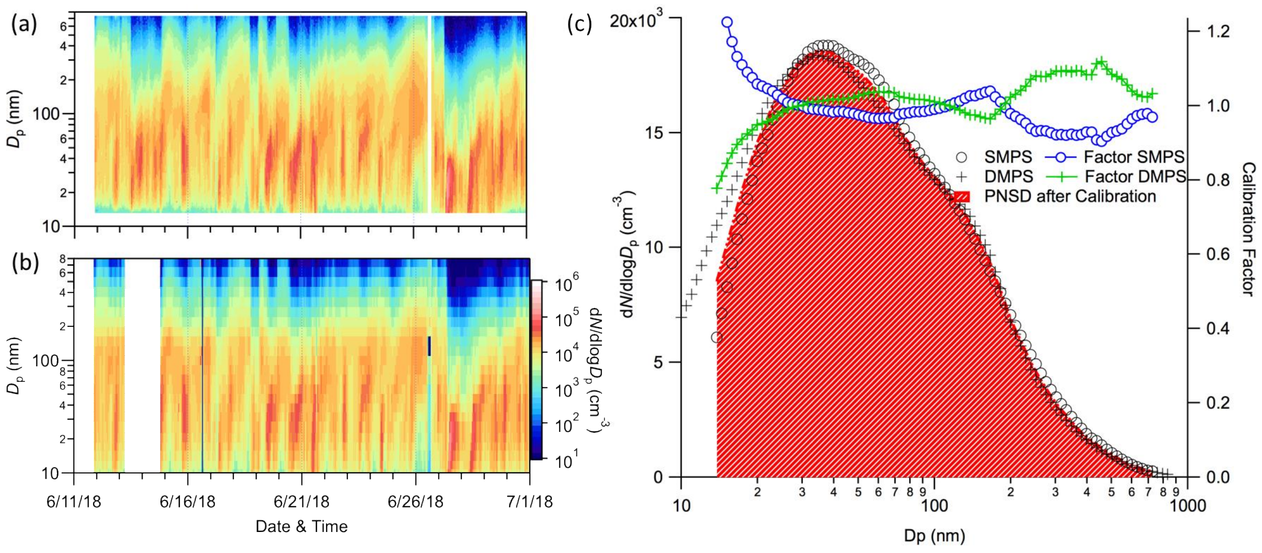

Figure S1. Particle number size distribution was measured during the comparison period from June 11th to July 1st, 2018 using (a) SMPS and (b) DMPS. (c) The average calibrated particle number size distribution was measured by SMPS and DMPS during the comparison period.

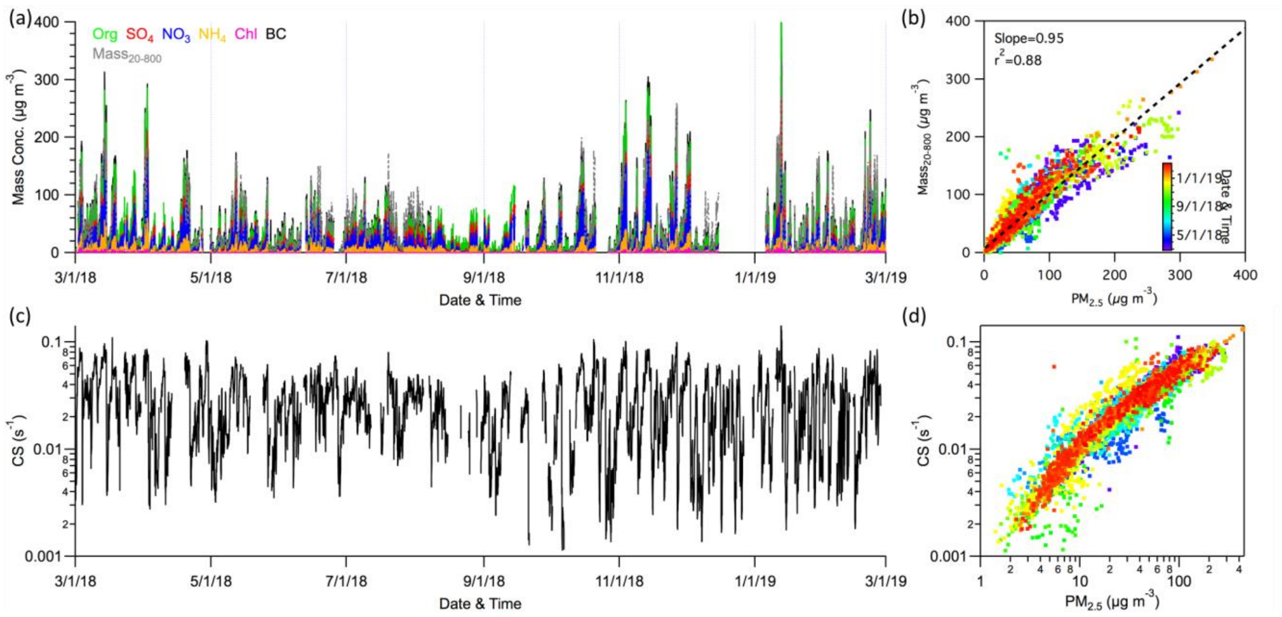

Figure S2. (a) The time series of mass concentration converted from SMPS ($Mass_{20\text{-}800}$), and chemical composition of fine particles ($PM_{2.5}$) measured by ACSM and AE33, including organic (Org), sulfate (SO_4), nitrate (NO_3), ammonium (NH_4), and chloride (Chl). (b) shows the comparison between $Mass_{20\text{-}800}$ and $PM_{2.5}$. (c) the time-series of condensation sink (CS) calculated based on the particle number size distribution. (d) shows the comparison between CS and $PM_{2.5}$.

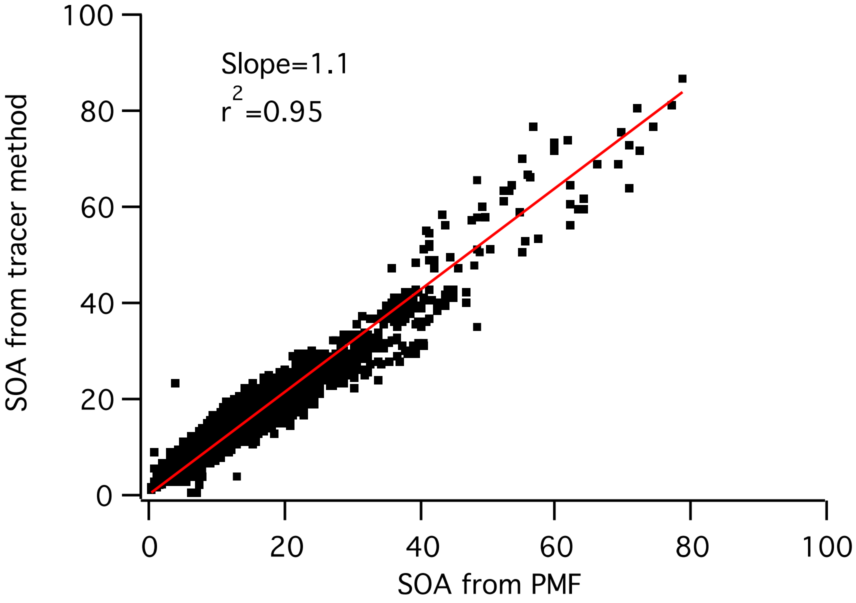

Figure S3. The comparison between secondary organic aerosols (SOA) from the tracer method and from the PMF method.

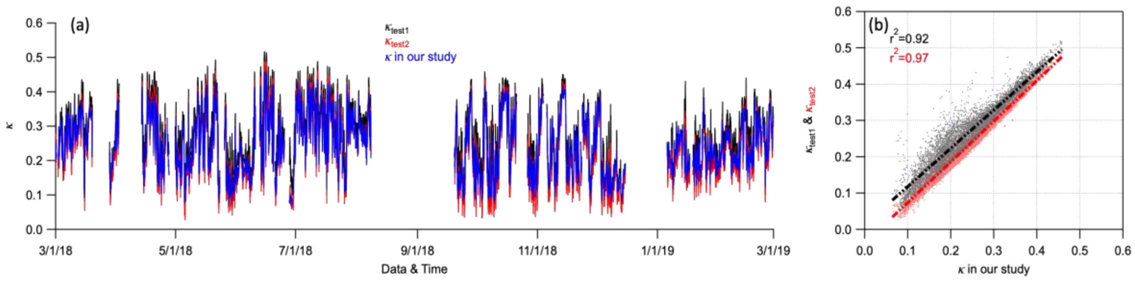

Figure S4. (a) and (b) are the time series and scatter plot of calculated κ_{test1} assuming secondary organic aerosol (SOA) has a κ_{SOA} value of 0.15, κ_{test2} assuming SOA has a κ_{SOA} value of 0.05, and κ in our study assuming organic aerosol has a constant κ_{org} value of 0.06 for organic aerosol.

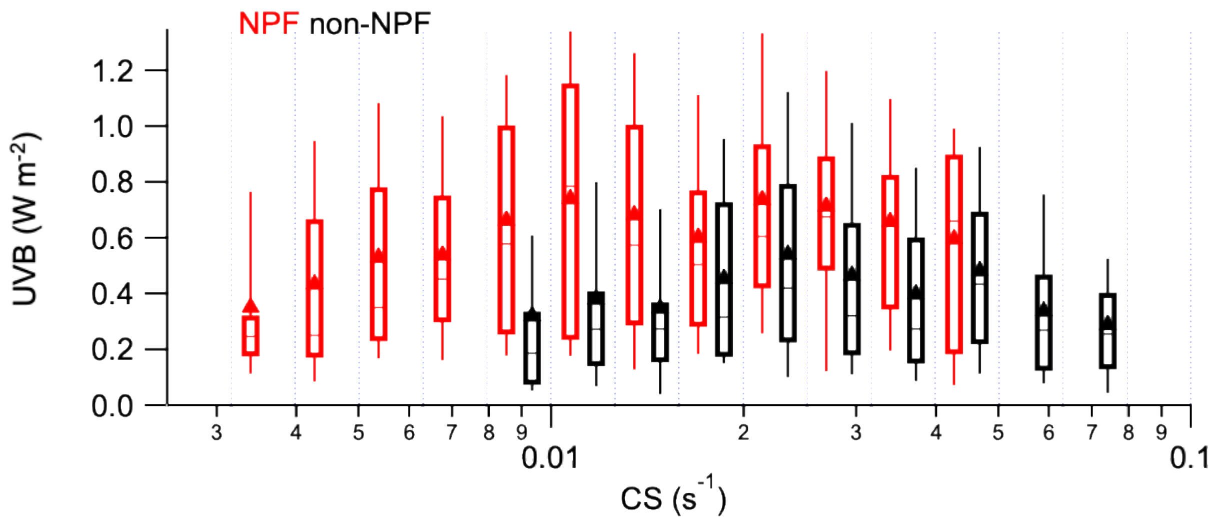

Figure S5. UVB as a function of CS during NPF (red) and non-NPF (black) days. Within each box, which corresponds to a logarithmic CS bin the median (middle horizontal line), mean (solid triangles), 25th and 75th percentiles (lower and upper ends of the box), and 10th and 90th percentiles (lower and upper whiskers) are shown.

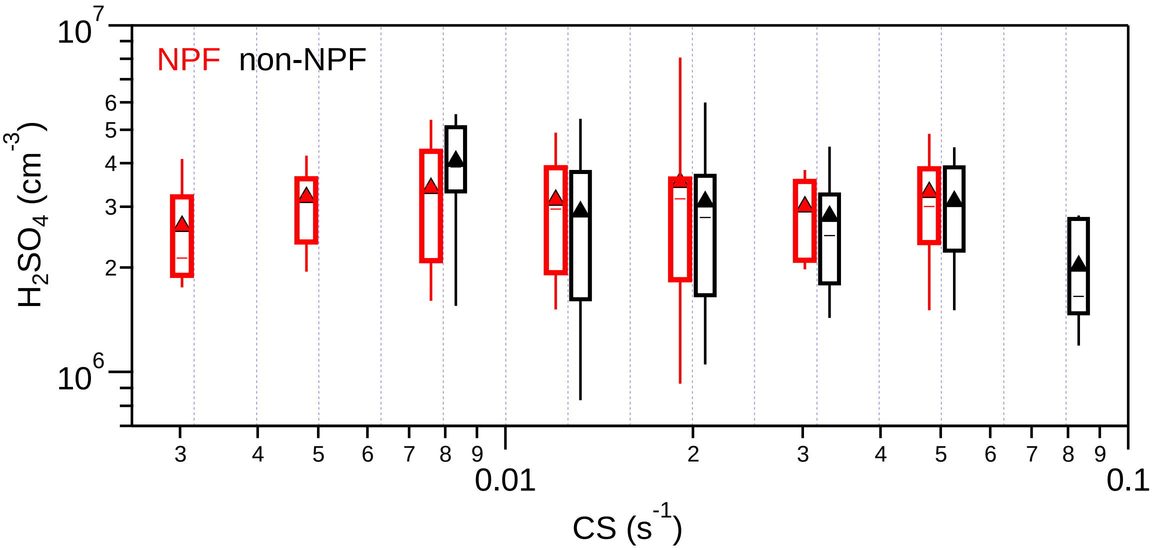

Figure S6. The concentration of sulfuric acid (H_2SO_4) as a function of CS during NPF (red) and non-NPF (black) days. Within each box, which corresponds to a logarithmic CS bin the median (middle horizontal line), mean (solid triangles), 25th and 75th percentiles (lower and upper ends of the box), and 10th and 90th percentiles (lower and upper whiskers) are shown.

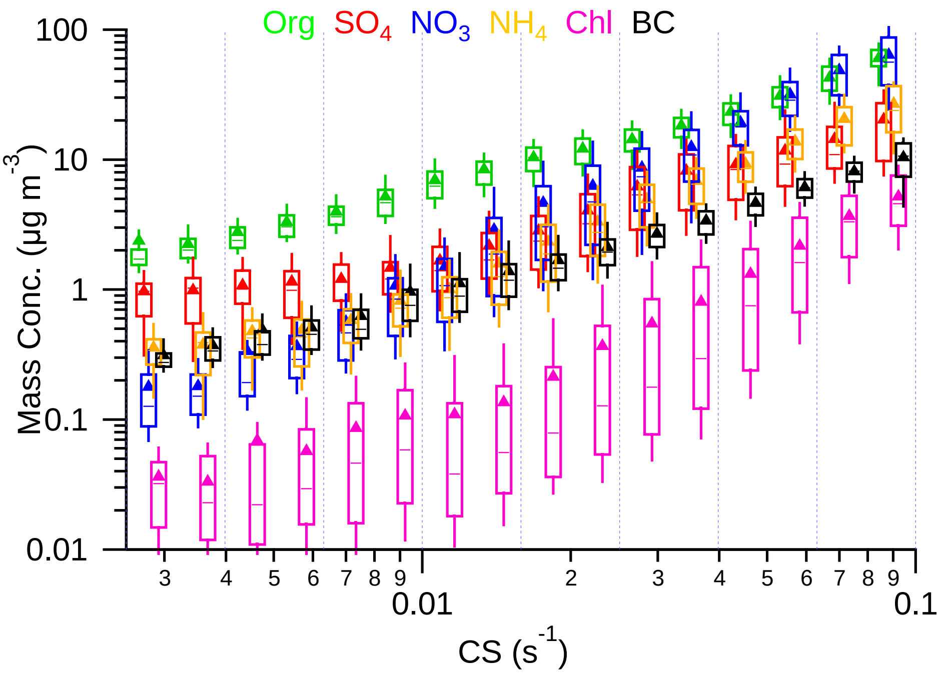

Figure S7. (a) Mass concentration of particle chemical components as a function of condensation sink (CS). Within each box, which corresponds to a logarithmic CS bin the median (middle horizontal line), mean (solid circles), 25th and 75th percentiles (lower and upper ends of the box), and 10th and 90th percentiles (lower and upper whiskers) are shown.

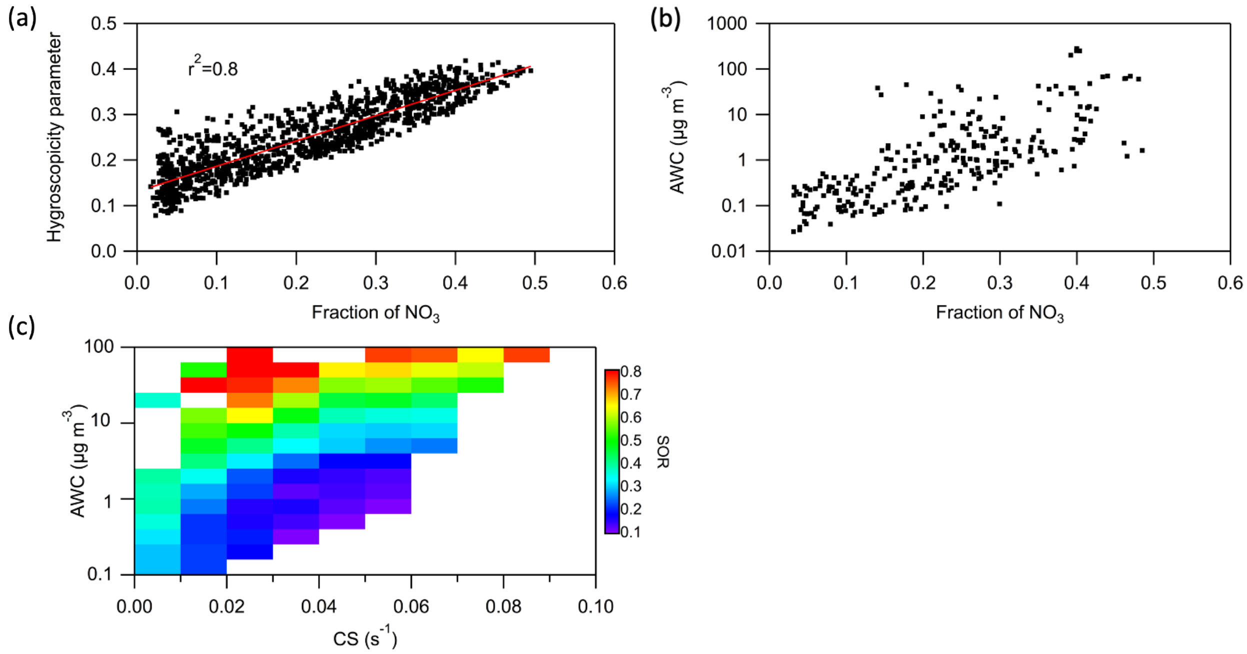

Figure S8. The scatter plot of (a) Hygroscopicity parameter (κ) and (b) aerosol water content (AWC) versus the fraction of nitrate in $PM_{2.5}$. (c) The variation of calculated aerosol water content (AWC) as the function of condensation sink (CS), which is colored by sulfur oxidation ratios (SOR).

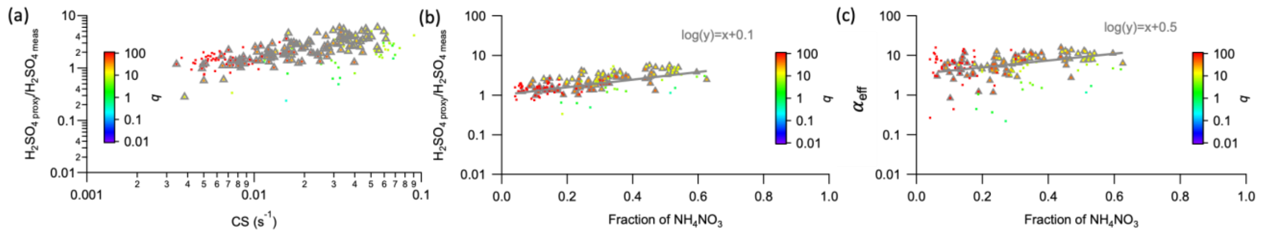

Figure S9. (a) and (b) show the evolution of the ratio between estimated H_2SO_4 (H_2SO_{4proxy}) and measured H_2SO_4 (H_2SO_{4meas}) concentration as a function of CS and mass fraction of ammonium nitrate during the daytime from 10:00 to 15:00, respectively. (c) is the correlation between α_{eff} and the fraction of ammonium nitrate. The color changed from purple to red with b in eq. 7 increasing from 0.01 to 100. The gray lines in (b) and (c) are linear fitting of typical daytime values with the triangle (10<b<100).

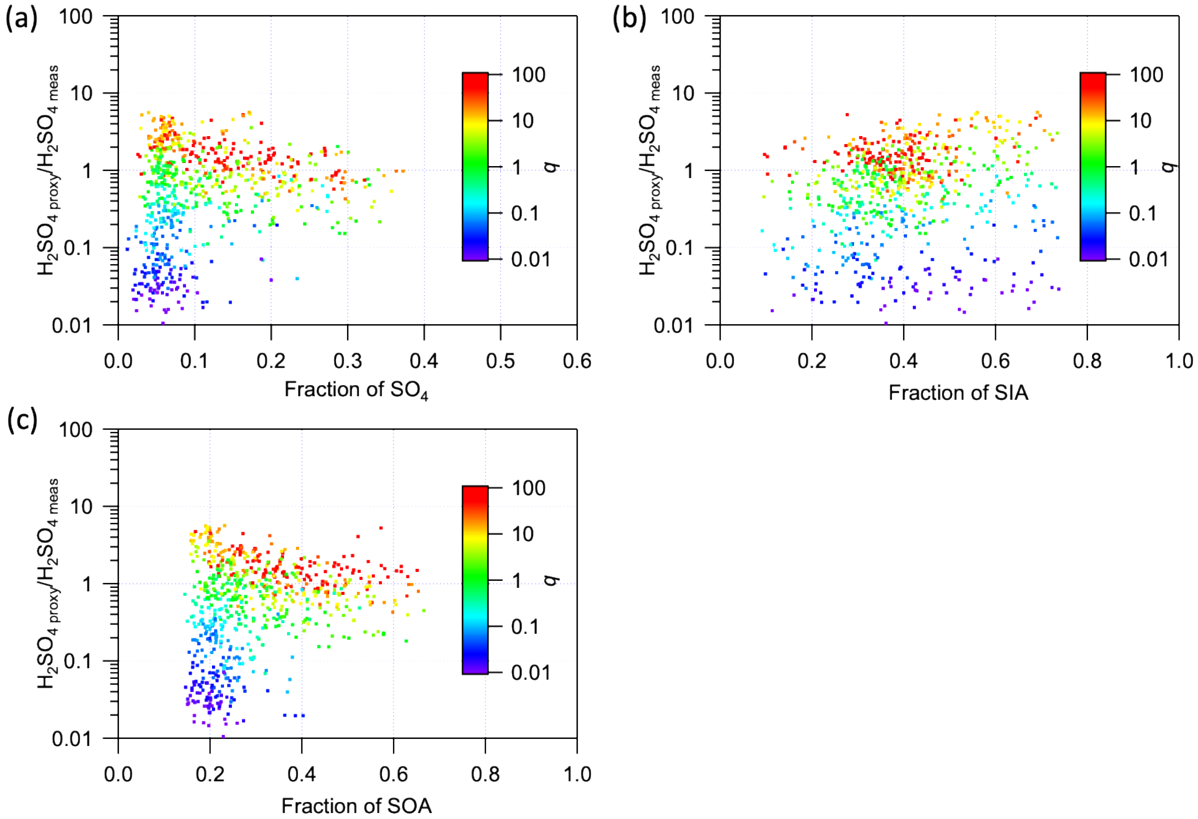

Figure S10. The evolution of the ratio between estimated H_2SO_4 (H_2SO_{4proxy}) and measured H_2SO_4 (H_2SO_{4meas}) concentration as a function of mass fraction of (a) Sulfate (SO_4), (b) secondary inorganic aerosols (SIA), and (c) secondary organic aerosols (SOA) during our observation. The color changed from purple to red with b increasing from 0.01 to 100.

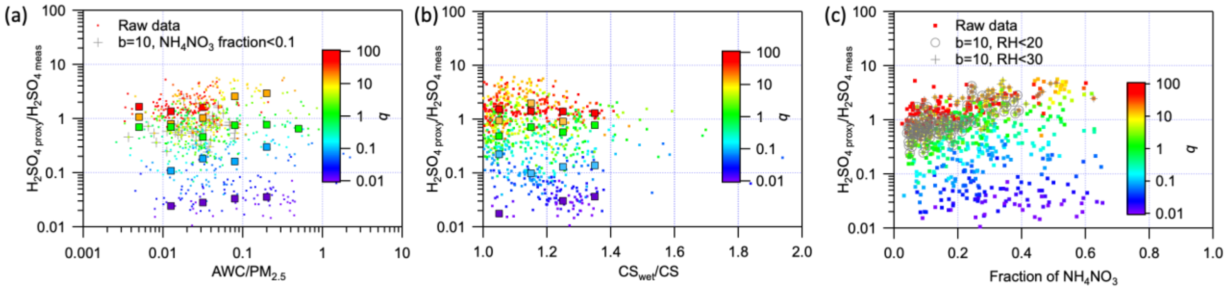

Figure S11. The scatter plot of the ratio between estimated H_2SO_4 (H_2SO_{4proxy}) and measured H_2SO_4 (H_2SO_{4meas}) concentration versus (a) the ratio of AWC to the $PM_{2.5}$ particle mass, (b) the ratio of the wet CS (CS_{wet}) to CS, (c) NH_4NO_3 fraction. Dots were colored by the *b* value. The median values after binned are also shown (squares).

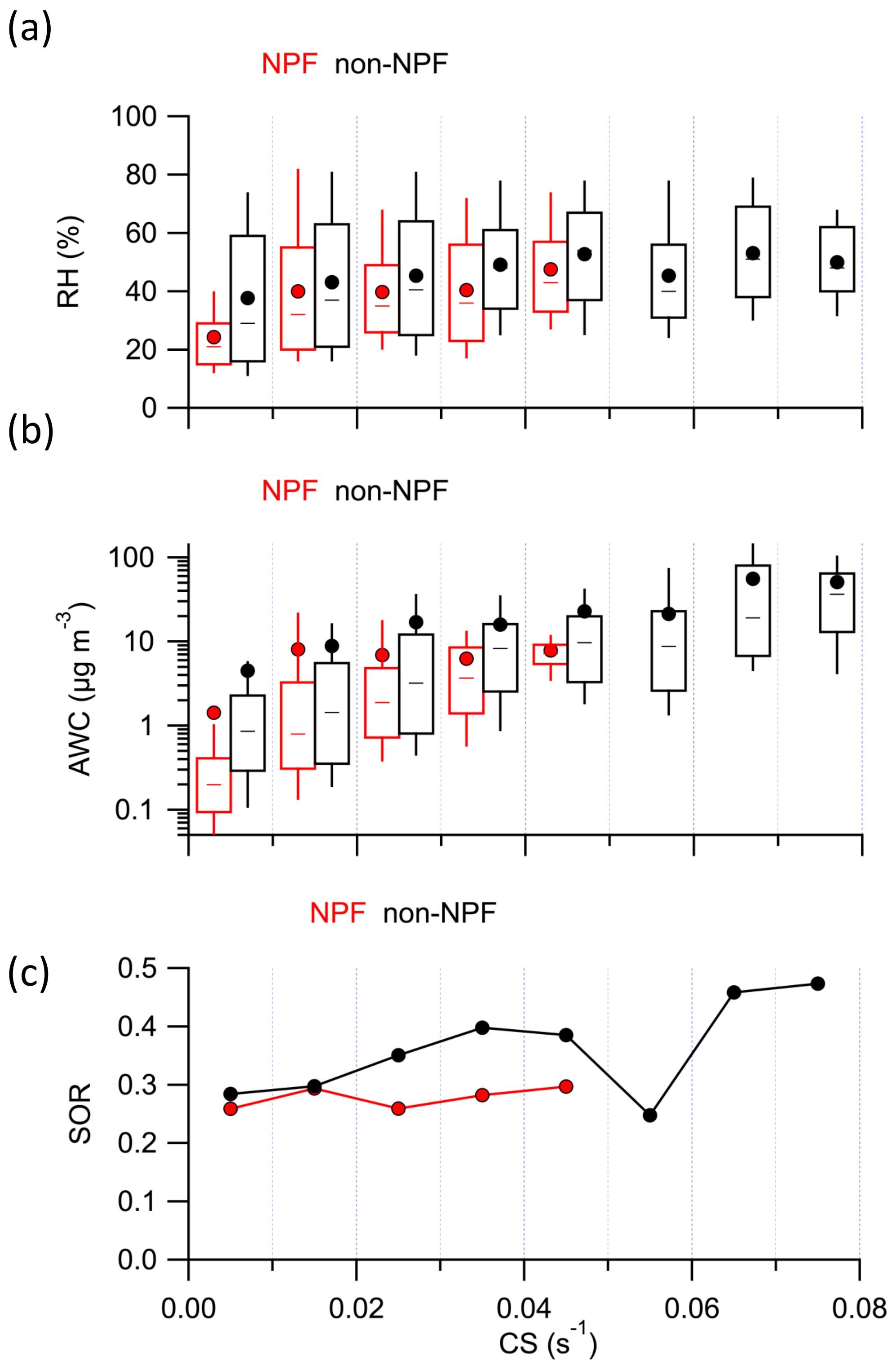

Figure S12. The evolution of (a) relative humidity (RH), (b) aerosol water content (AWC), (c) sulfur oxidation ratios (SOR) as a function of CS during NPF and non-NPF days, respectively. The median (middle horizontal line), mean (solid circles), 25th and 75th percentiles (lower and upper box), and 10th and 90th percentiles (lower and upper whiskers) are shown for each bin.

References

1. Wiedensohler, A.; Birmili, W.; Nowak, A.; Sonntag, A.; Weinhold, K.; Merkel, M.; Wehner, B.; Tuch, T.; Pfeifer, S.; Fiebig, M.; Fjäraa, A. M.; Asmi, E.; Sellegri, K.; Depuy, R.; Venzac, H.; Villani, P.; Laj, P.; Aalto, P.; Ogren, J. A.; Swietlicki, E.; Williams, P.; Roldin, P.; Quincey, P.; Hüglin, C.; Fierz-Schmidhauser, R.; Gysel, M.; Weingartner, E.; Riccobono, F.; Santos, S.; Grüning, C.; Faloon, K.; Beddows, D.; Harrison, R.; Monahan, C.; Jennings, S. G.; O'Dowd, C. D.; Marinoni, A.; Horn, H. G.; Keck, L.; Jiang, J.; Scheckman, J.; McMurry, P. H.; Deng, Z.; Zhao, C. S.; Moerman, M.; Henzing, B.; de Leeuw, G.; Löschau, G.; Bastian, S., Mobility particle size spectrometers: harmonization of technical standards and data structure to facilitate high quality long-term observations of atmospheric particle number size distributions. *Atmospheric Measurement Techniques* **2012,** *5*, (3), 657-685.
2. Cai, J.; Chu, B.; Yao, L.; Yan, C.; Heikkinen, L. M.; Zheng, F.; Li, C.; Fan, X.; Zhang, S.; Yang, D.; Wang, Y.; Kokkonen, T. V.; Chan, T.; Zhou, Y.; Dada, L.; Liu, Y.; He, H.; Paasonen, P.; Kujansuu, J. T.; Petäjä, T.; Mohr, C.; Kangasluoma, J.; Bianchi, F.; Sun, Y.; Croteau, P. L.; Worsnop, D. R.; Kerminen, V.-M.; Du, W.; Kulmala, M.; Daellenbach, K. R., Size-segregated particle number and mass concentrations from different emission sources in urban Beijing. *Atmospheric Chemistry and Physics* **2020,** *20*, (21), 12721-12740.
3. Ng, N.; Canagaratna, M.; Jimenez, J.; Zhang, Q.; Ulbrich, I.; Worsnop, D., Real-time methods for estimating organic component mass concentrations from aerosol mass spectrometer data. *Environmental Science & Technology* **2011,** *45*, (3), 910-916.
4. Kulmala, M.; Dada, L.; Daellenbach, K. R.; Yan, C.; Stolzenburg, D.; Kontkanen, J.; Ezhova, E.; Hakala, S.; Tuovinen, S.; Kokkonen, T. V., Is reducing new particle formation a plausible solution to mitigate particulate air pollution in Beijing and other Chinese megacities? *Faraday Discussions* **2021**.
5. Wang, Y. Q.; Zhang, X. Y.; Sun, J. Y.; Zhang, X. C.; Che, H. Z.; Li, Y., Spatial and temporal variations of the concentrations of PM10, PM2.5 and PM1 in China. *Atmospheric Chemistry and Physics* **2015,** *15*, (23), 13585-13598.
6. Zhang, Y.; Lang, J.; Cheng, S.; Li, S.; Zhou, Y.; Chen, D.; Zhang, H.; Wang, H., Chemical composition and sources of PM1 and PM2.5 in Beijing in autumn. *Science of the Total Environment* **2018,** *630*, 72-82.
7. DeCarlo, P. F.; Slowik, J. G.; Worsnop, D. R.; Davidovits, P.; Jimenez, J. L., Particle Morphology and Density Characterization by Combined Mobility and Aerodynamic Diameter Measurements. Part 1: Theory. *Aerosol Science and Technology* **2004,** *38*, (12), 1185-1205.
8. Jokinen, T.; Sipilä, M.; Junninen, H.; Ehn, M.; Lönn, G.; Hakala, J.; Petäjä, T.; Mauldin, R. L.; Kulmala, M.; Worsnop, D. R., Atmospheric sulphuric acid and neutral cluster measurements using CI-APi-TOF. *Atmospheric Chemistry and Physics* **2012,** *12*, (9), 4117-4125.
9. Kürten, A.; Rondo, L.; Ehrhart, S.; Curtius, J., Calibration of a chemical ionization mass spectrometer for the measurement of gaseous sulfuric acid. *The Journal of Physical Chemistry A* **2012,** *116*, (24), 6375-6386.
10. Yan, C.; Yin, R.; Lu, Y.; Dada, L.; Yang, D.; Fu, Y.; Kontkanen, J.; Deng, C.; Garmash, O.; Ruan, J.; Baalbaki, R.; Schervish, M.; Cai, R.; Bloss, M.; Chan, T.; Chen, T.; Chen, Q.; Chen, X.; Chen, Y.; Chu, B.; Dällenbach, K.; Foreback, B.; He, X.; Heikkinen, L.; Jokinen, T.; Junninen, H.; Kangasluoma, J.; Kokkonen, T.; Kurppa, M.; Lehtipalo, K.; Li, H.; Li, H.; Li, X.; Liu, Y.; Ma, Q.; Paasonen, P.; Rantala, P.; Pileci, R. E.; Rusanen, A.; Sarnela, N.; Simonen, P.; Wang, S.; Wang, W.; Wang, Y.; Xue, M.; Yang, G.; Yao, L.; Zhou, Y.; Kujansuu, J.; Petäjä, T.; Nie, W.; Ma, Y.; Ge, M.; He, H.; Donahue, N. M.; Worsnop, D. R.; Veli‐Matti, K.; Wang, L.; Liu, Y.; Zheng, J.; Kulmala, M.; Jiang, J.; Bianchi, F., The Synergistic Role of Sulfuric Acid, Bases, and Oxidized Organics Governing New‐Particle Formation in Beijing. *Geophysical Research Letters* **2021,** *48*, (7).

11. Liu, Y.; Zhang, Y.; Lian, C.; Yan, C.; Feng, Z.; Zheng, F.; Fan, X.; Chen, Y.; Wang, W.; Chu, B.; Wang, Y.; Cai, J.; Du, W.; Daellenbach, K. R.; Kangasluoma, J.; Bianchi, F.; Kujansuu, J.; Petäjä, T.; Wang, X.; Hu, B.; Wang, Y.; Ge, M.; He, H.; Kulmala, M., The promotion effect of nitrous acid on aerosol formation in wintertime in Beijing: the possible contribution of traffic-related emissions. *Atmospheric Chemistry and Physics* **2020,** *20*, (21), 13023-13040.
12. Wang, Y.; Clusius, P.; Yan, C.; Dallenbach, K.; Yin, R.; Wang, M.; He, X. C.; Chu, B.; Lu, Y.; Dada, L.; Kangasluoma, J.; Rantala, P.; Deng, C.; Lin, Z.; Wang, W.; Yao, L.; Fan, X.; Du, W.; Cai, J.; Heikkinen, L.; Tham, Y. J.; Zha, Q.; Ling, Z.; Junninen, H.; Petaja, T.; Ge, M.; Wang, Y.; He, H.; Worsnop, D. R.; Kerminen, V. M.; Bianchi, F.; Wang, L.; Jiang, J.; Liu, Y.; Boy, M.; Ehn, M.; Donahue, N. M.; Kulmala, M., Molecular Composition of Oxygenated Organic Molecules and Their Contributions to Organic Aerosol in Beijing. *Environmental Science & Technology* **2022,** *56*, (2), 770-778.
13. Petters, M. D.; Kreidenweis, S. M., A single parameter representation of hygroscopic growth and cloud condensation nucleus activity. *Atmospheric Chemistry and Physics* **2007,** *7*, (8), 1961-1971.
14. Gysel, M.; Crosier, J.; Topping, D. O.; Whitehead, J. D.; Bower, K. N.; Cubison, M. J.; Williams, P. I.; Flynn, M. J.; McFiggans, G. B.; Coe, H., Closure study between chemical composition and hygroscopic growth of aerosol particles during TORCH2. *Atmospheric Chemistry and Physics* **2007,** *7*, (24), 6131-6144.
15. Zhao, D. F.; Buchholz, A.; Kortner, B.; Schlag, P.; Rubach, F.; Kiendler-Scharr, A.; Tillmann, R.; Wahner, A.; Flores, J. M.; Rudich, Y.; Watne, Å. K.; Hallquist, M.; Wildt, J.; Mentel, T. F., Size-dependent hygroscopicity parameter (κ) and chemical composition of secondary organic cloud condensation nuclei. *Geophysical Research Letters* **2015,** *42*, (24), 10,920-10,928.
16. Wu, Z. J.; Zheng, J.; Shang, D. J.; Du, Z. F.; Wu, Y. S.; Zeng, L. M.; Wiedensohler, A.; Hu, M., Particle hygroscopicity and its link to chemical composition in the urban atmosphere of Beijing, China, during summertime. *Atmospheric Chemistry and Physics* **2016,** *16*, (2), 1123-1138.
17. Guo, H.; Xu, L.; Bougiatioti, A.; Cerully, K. M.; Capps, S. L.; Hite, J. R.; Carlton, A. G.; Lee, S. H.; Bergin, M. H.; Ng, N. L.; Nenes, A.; Weber, R. J., Fine-particle water and pH in the southeastern United States. *Atmospheric Chemistry and Physics* **2015,** *15*, (9), 5211-5228.
18. Xu, Y.; Miyazaki, Y.; Tachibana, E.; Sato, K.; Ramasamy, S.; Mochizuki, T.; Sadanaga, Y.; Nakashima, Y.; Sakamoto, Y.; Matsuda, K.; Kajii, Y., Aerosol Liquid Water Promotes the Formation of Water-Soluble Organic Nitrogen in Submicrometer Aerosols in a Suburban Forest. *Environmental Science & Technology* **2020,** *54*, (3), 1406-1414.
19. Yao, X.; Chan, C. K.; Fang, M.; Cadle, S.; Chan, T.; Mulawa, P.; He, K.; Ye, B., The water-soluble ionic composition of PM2. 5 in Shanghai and Beijing, China. *Atmospheric Environment* **2002,** *36*, (26), 4223-4234.
20. Dal Maso, M.; Kulmala, M.; Riipinen, I.; Wagner, R.; Hussein, T.; Aalto, P. P.; Lehtinen, K. E. J., Formation and growth of fresh atmospheric aerosols: eight years of aerosol size distribution data from SMEAR II, Hyytiala, Finland. *Boreal Environment Research* **2005,** *10*, (5), 323-336.
21. Zhou, Y.; Dada, L.; Liu, Y.; Fu, Y.; Kangasluoma, J.; Chan, T.; Yan, C.; Chu, B.; Daellenbach, K. R.; Bianchi, F.; Kokkonen, T. V.; Liu, Y.; Kujansuu, J.; Kerminen, V.-M.; Petäjä, T.; Wang, L.; Jiang, J.; Kulmala, M., Variation of size-segregated particle number concentrations in wintertime Beijing. *Atmospheric Chemistry and Physics* **2020,** *20*, (2), 1201-1216.
22. Chu, B.; Dada, L.; Liu, Y.; Yao, L.; Wang, Y.; Du, W.; Cai, J.; Dallenbach, K. R.; Chen, X.; Simonen, P.; Zhou, Y.; Deng, C.; Fu, Y.; Yin, R.; Li, H.; He, X. C.; Feng, Z.; Yan, C.; Kangasluoma, J.; Bianchi, F.; Jiang, J.; Kujansuu, J.; Kerminen, V. M.; Petaja, T.; He, H.; Kulmala, M., Particle growth with photochemical age from new particle formation to haze in the winter of Beijing, China. *Science of the Total Environment* **2021,** *753*, 142207.

23. Dada, L.; Chellapermal, R.; Buenrostro Mazon, S.; Paasonen, P.; Lampilahti, J.; Manninen, H. E.; Junninen, H.; Petäjä, T.; Kerminen, V.-M.; Kulmala, M., Refined classification and characterization of atmospheric new-particle formation events using air ions. *Atmospheric Chemistry and Physics* **2018,** *18*, (24), 17883-17893.
24. Dada, L.; Ylivinkka, I.; Baalbaki, R.; Li, C.; Guo, Y.; Yan, C.; Yao, L.; Sarnela, N.; Jokinen, T.; Daellenbach, K. R.; Yin, R.; Deng, C.; Chu, B.; Nieminen, T.; Wang, Y.; Lin, Z.; Thakur, R. C.; Kontkanen, J.; Stolzenburg, D.; Sipilä, M.; Hussein, T.; Paasonen, P.; Bianchi, F.; Salma, I.; Weidinger, T.; Pikridas, M.; Sciare, J.; Jiang, J.; Liu, Y.; Petäjä, T.; Kerminen, V.-M.; Kulmala, M., Sources and sinks driving sulfuric acid concentrations in contrasting environments: implications on proxy calculations. *Atmospheric Chemistry and Physics* **2020,** *20*, (20), 11747-11766.
25. Shen, C.; Zhao, G.; Zhao, W.; Tian, P.; Zhao, C., Measurement report: aerosol hygroscopic properties extended to 600 nm in the urban environment. *Atmospheric Chemistry and Physics* **2021,** *21*, (3), 1375-1388.
26. Holmgren, H.; Sellegri, K.; Hervo, M.; Rose, C.; Freney, E.; Villani, P.; Laj, P., Hygroscopic properties and mixing state of aerosol measured at the high-altitude site Puy de Dôme (1465 m a.s.l.), France. *Atmospheric Chemistry and Physics* **2014,** *14*, (18), 9537-9554.
27. Ehn, M.; Petäjä, T.; Aufmhoff, H.; Aalto, P.; Hämeri, K.; Arnold, F.; Laaksonen, A.; Kulmala, M., Hygroscopic properties of ultrafine aerosol particles in the boreal forest: diurnal variation, solubility and the influence of sulfuric acid. *Atmospheric Chemistry and Physics* **2007,** *7*, (1), 211-222.
28. Chen, L.; Zhang, F.; Zhang, D.; Wang, X.; Song, W.; Liu, J.; Ren, J.; Jiang, S.; Li, X.; Li, Z., Measurement report: Hygroscopic growth of ambient fine particles measured at five sites of China. *Atmos. Chem. Phys. Discuss.* **2022,** *2022*, 1-28.
29. Kulmala, M.; Kerminen, V. M.; Petaja, T.; Ding, A. J.; Wang, L., Atmospheric gas-to-particle conversion: why NPF events are observed in megacities? *Faraday Discussions* **2017,** *200*, 271-288.
